# Biopolymers and Biomaterials for Special Applications within the Context of the Circular Economy

**DOI:** 10.3390/ma14247704

**Published:** 2021-12-13

**Authors:** Radosław Dziuba, Magdalena Kucharska, Longina Madej-Kiełbik, Konrad Sulak, Maria Wiśniewska-Wrona

**Affiliations:** 1 Department of World Economy and European Integration, University of Lodz, 41/43 Rewolucji 1905 Str., 90-214 Lodz, Poland; radoslaw.dziuba@uni.lodz.pl; 2 Łukasiewicz Research Network—Institute of Biopolymers and Chemical Fibres, 19/27 M. Sklodowskiej-Curie Str., 90-570 Lodz, Poland; magdalena.kucharska@ibwch.lukasiewicz.gov.pl (M.K.); longina.madej-kielbik@ibwch.lukasiewicz.gov.pl (L.M.-K.); k.sulak@ibwch.lukasiewicz.gov.pl (K.S.)

**Keywords:** economy, materials engineering, circular economy, biomaterials, biopolymers, biodegradable polymers

## Abstract

The main challenge of the economy is counteracting the adverse effects of progressive industrialisation on the environment around the world. Economic development that accompanies this trend correlates to production increase in not only consumer articles but also special application articles that are difficult to remanufacture, such as medical supplies. For many researchers, discovering innovative materials for special applications that could become an essential element of circular economy production is important. Measures to reduce the production of industrial materials whose waste is difficult to recycle are more and more apparent to manufacturers, especially when faced with the new financial situation in European Union, as one of its priorities is to implement the principles of circular economy. The purpose of the article is to analyse the current state of research on special-application biomaterials within the context of the circular economy. Empirical analysis is conducted for Poland compared to the rest of the European Union (EU) within the time-frame of 2014–2020, which is the most recent financial timeframe of the EU. The submitted studies are based on secondary data obtained mainly from European databases, as well as primary data resulting from the research works at Łukasiewicz Research Network—Institute of Biopolymers and Chemical Fibres.

## 1. Introduction

**Figure 1:**
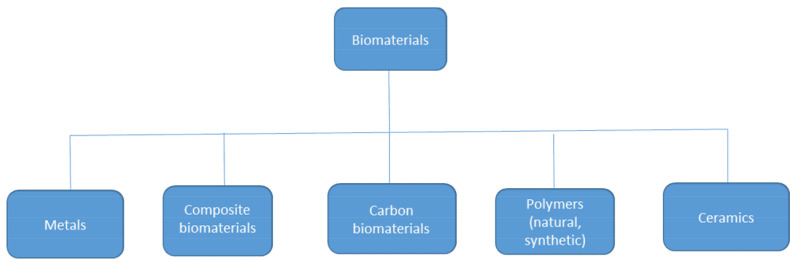
General division of biomaterials.

Biomaterials, which can be alive or lifeless, include both natural and synthetic polymers, composites, ceramics, and metals, are defined as materials that interact with biological systems [1]. The general division of biomaterials is presented in Figure 1. The framework of fully interactive, biocompatible, biodegradable, and non-cytotoxic biological systems is still widely used in various areas of life [2]. Currently, polymeric materials are widely used in many industries. Their global production is expected to exceed 500 million tons by 2050 [3]. At present, some discarded polymeric materials have accumulated in the environment due to their high durability. The management of plastic waste mainly consists of dumping in landfills and incineration [4]. The landfill sites are a source of groundwater contamination, and the incineration of waste materials results in hazardous gas emissions [5]; therefore, the selection of an appropriate polymer enables the production of innovative materials that may become the basic element of production within the framework of the principles of the circular economy.

The bioeconomy connects the economy, science, industry, and society. The great challenges for bioeconomies span from resource-efficient manufacturing of products such as chemicals, materials, food, pharmaceuticals, polymers, flavourings, and fragrances to the production of new biomaterials and bioenergy in a sustainable and economic way to meet the needs of the continuously growing world population [6]. Biomaterials are increasingly replacing traditional polymeric materials; hence, they can be used for special applications. In line with the principles of the circular economy, more and more attempts are made to substantially reduce the production of materials used in various industries, which are at the same time the source of waste that is difficult to reprocess. The growing interest in modern polymer materials triggers the gathering of up-to-date scientific information on the complex chemical structure of plastics and the possibility of modifying their properties during processing, which may be useful for specialists from various fields of science. Besides the medical industry, the textile industry is an important branch that uses polymer materials. Production in textile plants generates a wide variety of solid and liquid wastes that have to be properly managed. Some of them can be recycled, while others are incinerated or landfilled. In some cases, the waste can be processed in fermentation chambers. More and more often, manufacturing companies that use polymer materials are obliged to systematically adapt their methods of planning and production to the requirements of sustainable development so that a high level of environmental protection is ensured on the whole through the integrated pollution-prevention process as much as possible, where it is not fully possible to reduce pollution. The main assumption of the European Strategy for Plastics in a Circular Economy relies on an ambitious approach for the recycling of plastic materials and emphasises the importance of preventing microplastic release, which currently is one of the most widespread sources of marine pollution [6]. Nature has also suffered from the COVID-19 pandemic according to a report released by the European Environment Agency. The increased production and use of medical/protective masks has resulted in greater emissions of greenhouse gases and other harmful gases and has generated waste that can harm animals and entire ecosystems. Scientists have estimated that during the pandemic, the human population is using approx. 3 million disposable masks per minute, which amounts to as many as 130 billion masks per month. Assuming that a disposable surgical mask weighs approx. 3.5 g, it turns out that the total weight of masks used every month exceeds 450 t. Therefore, more and more emphasis is placed on the use of biomaterials, including biopolymers, in various branches of life. Additionally, in the Łukasiewicz Research Network at the Institute of Biopolymers and Chemical Fibres, research is conducted on the use of biodegradable polymers as an alternative to the polypropylene materials currently used in industry. Biodegradable polymers may help to reduce the costs associated with the disposal or storage of waste and, at the same time, constitute a response to global trends in the production of different, mainly disposable products. The aim of the circular economy is to treat wastes as valuable raw material flowing into the industrial economy and incorporate them into the life cycle of subsequent products, i.e., the flow of non-renewable raw materials in closed circuits. The unsustainable management of natural resources and wastes leads to adverse effects, the scale of which is sometimes difficult to estimate. Wastes are more and more often improperly managed, and therefore the scale of their negative impact on the environment is increasing. The substitution of traditional plastics with biomaterials and biopolymers is a response to Directive (EU) 2019/904 of the European Parliament and of the Council the European Union of 5 June 2019 [7] on reducing the environmental impact of certain plastic products. The products mentioned in the directive can be replaced by products made from alternative biodegradable raw materials. In May 2018, a package of directives on waste was implemented that imposes new obligations on the member states in the field of waste management and disposal and sets new levels of recycling. The new regulations establish the EU policy of waste management in the circular economy, and their main purpose is to prevent waste generation and, when this is not possible, to support its recycling and recovery. According to these regulations, the landfilling of waste should be systematically reduced, and its processing and re-use should become an increasingly common solution in order to reduce the negative impact of plastic waste on the environment. So far, most companies have been using raw materials from crude oil, but this is starting to change. Over the past few years, many companies have been choosing to invest in bio-based materials research, looking for ways to integrate more circular economy practices into their manufacturing processes. The circular economy is one of the key goals of many companies’ strategies, even though it is not an easy change. The assumption of the circular economy is to follow a certain hierarchy in which the priority is to prevent waste generation at the product design stage, minimise the consumption of materials and energy during production and use processes, avoid the use of substances that are potentially dangerous for the consumer and the environment, and subsequently to change the way products are manufactured and after their use to give them “another life” as a result of repair or re-use [8]. Replacing plastics with biopolymers is in line with the assumptions of circular economy, allowing the reduction of substances hazardous for the consumer and the environment. The use of plastics for the production of everyday use items causes a significant increase in environmentally harmful waste. The scale of the problem is presented by Bruno et al. [9] for the example of electronic waste, the level of which has increased significantly in recent years. Authors point to aspects related to the recovery, reuse, recycling, and disposal of electrowaste. Economic and environmental opportunities are mainly related to the overall value of the raw materials included in e-waste, which have a high recycling potential [10], while the environmental aspects lie in the possibility of minimising the environmental footprint of primary production, from raw material extraction to logistics and production processes [11]. However, currently only a small fraction of waste electrical and electronic equipment in the world is properly collected and recycled. According to the authors, the transition to the circular economy model can be accelerated by designing and implementing appropriate systems and practices for a closed supply chain of waste electrical and electronic equipment. However, according to the provisions of the relevant European directives, which specify only general requirements for the organisation of national collection systems, adopting specific policies and achieving the set goals is the responsibility of individual member states. The Waste Electrical and Electronic Equipment (WEEE) Directive (2002/96/EC), referred to in [9], sets targets to stimulate investments in effective and efficient national collection systems for waste electrical and electronic equipment. Studies have shown that more advanced countries have achieved their goals with ease, while less advanced countries have faced serious difficulties. Based on the requirements for medical devices, it can be assumed that not every recovered material can be reused for the production of medical devices; therefore, in the medical sector, an alternative may be biopolymers that are not harmful to the environment.

## 2. Smart Biopolymers for Special Applications

**Figure 2:**
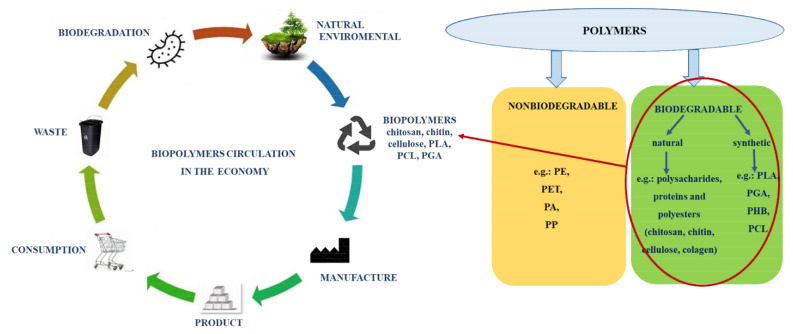
Life cycle of biodegradable polymers within the context of circular economy.

Biopolymers are biodegradable polymers produced from living organisms (such as microorganisms and plants). These polymers occur naturally in our body, but they can also be chemically synthesised from fats, amino acids, proteins, or vegetable oils, hence the division into natural and synthetic polymers (Figure 2).

Biodegradable polymers degrade to low-molecular-weight compounds under the action of micro- and/or macroorganisms or enzymes. Biodegradable polymers such as polycaprolactone (PCL) and polylactide (PLA), amide-containing polymers, polyurethane, and almost all natural polymers have heteroatoms in the main chain, which are susceptible to the hydrolytic cleavage of ester groups (–COO–), amide bonds (–CONH–), or ether bonds (–O–) [12]. Among the biodegradable materials, aliphatic polyesters such as PLA, polyhydroxyalkanoates (PHA) [13], PCL, poly (3-hydroxybutyrate) [14], and their corresponding copolymers are of great importance. Currently, the global bioplastics market, whose value is estimated at several billion dollars per year, is characterised by a dynamic growth rate [15]. Biodegradable polymers have numerous applications in different areas of life. Macromolecules that can be directly acquired from nature or are produced by organisms are also biodegradable polymers. These are, for example, natural rubber, lignin, and polysaccharides (cellulose, chitosan, chitin, sodium alginate, etc.). Lignin, found in plants, is still an ongoing challenge for scientists seeking ways to better utilise this abundant natural resource [16]. As can be seen in the literature [17], it is possible to develop a technically and eco-nomically feasible as well as sustainable and ecological process for the production of colloidal lignin on an industrial scale. Colloidal lignin can potentially replace petroleum-based raw materials such as polyethylene (PE), polypropylene (PP), and polyethylene terephthalate (PET) and can be used in various applications such as phenol formaldehyde (PF) resins, foams, carbon fillers, bactericides, and composites [18]. The more important polysaccharide, which has been widely used in medicine as a hydrogel preparation or alginate fibre for haemostatic gauze, is a sodium alginate [19]. Biodegradable polymers are widely used in many industries, ranging from medicine, food packaging, and agricultural mulch to electronics, including keyboards, and parts for the automotive sector [14]. Experimental results proved that biodegradable films compared with traditional PE agricultural mulch ensure a high crop yield and also do not cause soil pollution [20]. A serious problem for the environment is the ever-increasing industrial oily wastewater, the treatment of which has become a big worldwide problem [21]. As reported in the literature [22,23], conventional polyurethane foam can be replaced by highly porous polyester materials with exquisite oil-water separation properties due to their high porosity and strong hydrophobic properties. In addition, traditional polymers such as PP, Polystyrene (PS), and PE can be replaced by PLA, especially in the agricultural aspect. Every day, the use of portable electronics, such as smart sensors for health monitoring, is gaining popularity [24], as well as in wireless communications [25] and smart packaging [26]. The frequent upgrading of electronic equipment also brings the serious problem of electronic waste, which results in the pollution of the environment. Therefore, biodegradable polymers used for the production of flexible devices are becoming more and more popular. According to Gao et al. [27], transparent cellulose nanopaper and PLA electret can be used to create a biodegradable and transparent nanogenerator. Such a nanogenerator can be combined with paper to make a self-powered smart packaging system without compromising its appearance, due to the high transparency and desired output efficiency. As can be seen, such eco-friendly electronics may be an adequate solution to address the ongoing problem. The biodegradable materials currently used are based on polymers that have the ability to degrade under the influence of microorganisms. Contrary to polymers of petrochemical origin, whose decomposition periods range from 500 to even 1000 years, biodegradable polymers can totally degrade in just six months. There are fully biodegradable polymers that are completely converted into carbon dioxide, water, and organic substances, as well as compostable polymers that are degraded to carbon dioxide, water, inorganic compounds, and biomass [28]. Biodegradable materials together with materials produced on the basis of renewable raw materials, constitute so-called bioplastics. There are many types of biomaterials available on the market. The most popular biodegradable polymers distinguished by source are presented below.

### 2.1. Polymers from Renewable Resources

#### 2.1.1. Poly(Lactic Acid) PLA

The most technologically advanced biodegradable polymer is obtained from the natural monomer—lactic acid. Polylactide is commercially known under the following trade names: Lacea^®^ (Mitsui Toatsu, Tokyo, Japan), Lucty^®^ (Shimazu, Japan), and NatureWorks^®^ (Cargill Dow, Minnetonka, MN, USA). PLA can be obtained in many ways. First, the base, i.e., lactic acid, can be synthesised either chemically or biologically. The preferred method is biological synthesis, yielding two optically active enantiomers: L (+) or D (−), based on the bacterial fermentation of starch and other readily available carbohydrates derived from corn, sugar beet, sugar cane, or potatoes. A racemic mixture of L- and D-isomers is obtained by chemical synthesis from renewable intermediates (acetaldehyde, ethanol) or intermediates obtained from coal (acetylene) or crude oil. The polycondensation of lactic acid, depending on the technology used, leads to PLA with low or high molar mass. PLA can also be obtained by ring-opening polymerisation of the cyclic lactide. Since lactic acid exists in the form of L (+) or D (−) enantiomers, the lactide obtained may exist in the form of three stereoisomers: DD, LL, and DL. The properties of PLA depend on its stereochemical composition. The literature data show that the higher the degree of crystallinity, molar mass, and melting point of PLA, the slower the biodegradation process. L-oligomers are also more prone to degradation as opposed to racemic oligomers. Studies comparing the rate of degradation of some polyesters (PLA, polyhydroxybutyrate (PHB), PCL, polybutylene succinate (PBS) in soil show that among them, PLA is the least susceptible to microbial attack in a natural environment. A long period of time is necessary to start the degradation of PLA, which still is slow thereafter. Under the conditions of high temperature (50–60 °C) and high humidity prevailing in the compost, the decomposition of PLA into mono- and oligomers took 45–60 days. PLA fibre, in contrast to the commonly used PET, has a soft feel, just like wool and cotton. Due to the low water absorption, it is used in the production of sportswear. It is characterised by low flammability and does not emit smoke. The low density makes PLA lightweight. PLA is also resistant to ultraviolet radiation; hence, it is suitable for the production of outdoor furniture and equipment. The low refractive index of the polymer ensures perfect colour rendering. Due to the thermoplasticity, good mechanical properties, biodegradability, and non-toxicity of both the polymer and its decomposition products, PLA has been used in medicine as well as in the pharmaceutical and packaging industries. Initially, PLA has found application mainly in medicine, including controlled drug delivery and release systems and hydrogels, as a material for the production of orthopaedic screws, and in tissue engineering.

#### 2.1.2. Thermoplastic Starch TPS

Polymer–starch blends are used for the production of flexible and rigid films, trays, containers, foamed loose-fill filling up the voids in transport packaging, rigid injection-moulded packaging, as well as coatings for paper and cardboard. The following polymer–starch blends are available on the market: with polyester—(PCL) Mater-Bi^®^ (Novamont, Novara, Italy), Ecostar^®^ (National Starch, Bridgewater, NJ, USA), and Bioflex^®^ (Bio-Tech, Lexington, TX, USA), and with poly(vinyl alcohol)—Novoton^®^ (Chisso Corp. and Warner Lambert, Philadelphia, PA, USA).The addition of starch can lower the production cost of the material by, for instance, about 50% by replacing synthetic polyester with starch, which is more than 2.5 times cheaper.

#### 2.1.3. Polyesters of Microbiological Origin—3-hydroxybutyrate (PHA), poly(3-hydroxybutyrate-co-3-hydroxyvalerate) (PHBV), and poly(3-hydroxybutyrate-co-3-hydroxyhexanoate) (PHBH)

“Bacterial” biopolyesters are produced from living microorganisms. Zeneca Bioproducts (Billingham, UK) has developed a fermentation process that produces PHA copolymers. Among them, poly[(R)-3-hydroxybutyrate] [P (3HB)] and its copolymer with (R)-3-hydroxyvalerate [P (3HV)], known under the trade name Bio-pol^®^, are produced on an industrial scale. Another commercially produced biopolymer is a copolymer of (R)-3-hydroxybutyric acid with (R)-3-hydroxyhexanoic acid P (3HB-co-3HHx) known under the tradename Nodax™, manufactured by P&G-Kaneka (Osaka, Japan). Such bio-polyesters are used in agriculture and medicine. Polyester products in the form of fibres, films, bottles, and containers are degradable in soil and seawater. They are also used in medicine for the production of coatings for drugs of controlled release and bone implants, as well as in tissue engineering.

#### 2.1.4. Renewable Polyolefins

The main representative of this group of bioplastics is biopolyethylene (Bio-PE). In the production of this biopolymer petrochemical raw materials are replaced with raw materials from renewable resources.

### 2.2. Polymers Derived from Fossil Raw Materials

#### 2.2.1. Synthetic Aliphatic Polyesters

Polycaprolactone (PCL) is a polymer used to make biodegradable surgical sutures. Since its degradation time spans several months, it is used in the form of copolymers to increase the degradation rate. Due to the low softening point, it has also been used in the production of stiffening dressings. It is manufactured under the tradenames Tone^®^ (Union Carbide, Seadrift, TX, USA) and CAPA^®^ (Solvay, Beveren, Belgium) and Placeel^®^ (Daicel Chemical Ltd., Osaka, Japan).

Poly(butylene succinate) (PBS) is obtained from succinic acid and 1,4-butanediol, both of which in turn are produced from maleic anhydride. PBS is known as Bionolle^®^ (Showa Highpolymer, Tokyo, Japan) or SkyGreen BDP^®^ (SK Polymers, Seoul, Korea).

Polyglycolide (PGA) is the simplest aliphatic polyester. It was first used in 1960 to make fully synthetic resorbable sutures, known as Dexon™ (Davis and Geck, Inc., Brooklyn, NY, USA).

#### 2.2.2. Synthetic Aliphatic–Aromatic Copolymers

Aliphatic polyesters are more prone to degradation but have lower mechanical strength than their aromatic counterparts. In order to improve these properties, other monomers (aliphatic or aromatic) are incorporated in the chain of biodegradable aliphatic polyesters.

Poly(1,4-butylene adipate 1,4-butylene terephthalate) (PBAT) can be processed by the extrusion blow moulding method and, unlike PLA, it does not require drying before processing. It is produced under the trade name Ecoflex (BASF, Ludwigshafen, Germany).

Poly(trimethylene terephthalate) (PTT) has properties comparable to PET. Its use was initially limited by the low availability of one of the monomers. The flexibility and mechanical properties of PTT make this polymer suitable for the production of fibres.

### 2.3. Water-Soluble Polymers

Poly(vinyl alcohol) (PVA) is a polymer soluble in water and insoluble in most organic solvents. On a base of PVA, hydrogels are produced to be used in the production of membranes, active dressings, implants or in systems of controlled active substance release. Among the bioplastics, the most popular is the so-called Bio PET, i.e., a classic polymer material obtained, however, from renewable raw materials (of plant origin). On the other hand, PLA is the most popular among biodegradable plastics (approx. 38% of the production capacity of the above-mentioned polymers). The term “biodegradation” is sometimes defined ambiguously. In many countries, in order to standardise the nomenclature, technical standards are introduced, the fulfilment of which allows the producer of polymers or final products to label them as biodegradable [29].

## 3. Biomaterials for Special Applications Based on Natural Polymers

In the medical sector, biomaterials constitute the largest market segment, related mainly to the production of specialised dressing materials intended for the treatment of chronic and non-healing wounds. It offers dressing materials based mainly on natural polymers produced, among others, in the form of hydrogels and films [30,31]. It is estimated that by 2026, this market will have reached USD 16 billion, most of which will be in USA and European markets. It is closely related to the costs of treating difficult-to-heal wounds. Currently, the overall expenditure on wound care is USD 13–15 billion a year. In the European Union, it accounts for 2–4% of the health budget [32]. In Poland, the number of people suffering from so-called difficult wounds is estimated at over half a million [33]. Statistic data also show that, in Poland, about 100,000 people have wounds infected with Staphylococcus aureus, which are difficult to heal [34]. Worldwide, about 2% of people suffer from pressure ulcer wounds. Only in the European Union does expenditure on the treatment of difficult-to-heal wounds account for 2–4% of the health budget. In reference to the above-mentioned data for the Polish market, and assuming that, according to the financial plan for 2020, the Polish National Health Fund (NHF) budget amounted to PLN 90.7 billion, a 2% share of expenditure on wound treatment in Poland represents about PLN 1.8 billion per year. A significant part of this amount can be allocated directly to wound dressings [35]. Natural polymers with interesting biological properties derived from renewable sources include chitin and its derivative chitosan, which are the basic building blocks of the shells of marine crustaceans (e.g., crabs, shrimps, krill), and they are also produced by microbial fermentation from some species of bacteria or fungi [36,37]. Especially chitosan, due to its versatile properties, has been widely used in medicine, e.g., for the construction of dressing materials and implants, in drug delivery systems, in the production of dietary supplements, in cosmetics and antimicrobial fluids, and in plant cultivation and environmental protection processes, including wastewater treatment [38,39]. Numerous studies have proven its biodegradability and biocompatibility, as well as its lack of mutagenic properties and toxicity [40]. Moreover, the presence of two types of reactive functional groups—hydroxyl and amine moieties—gives the possibility of many different chemical and enzymatic modifications in order to create systems of immobilisation and cause the release of biologically active substances; they are also used for the preparation of water-soluble chitosan derivatives [41,42]. The antibacterial and antifungal activity of chitosan is attributed to the presence of positively charged amino groups, which interact with negatively charged lipopolysaccharides and proteins of microorganisms by disintegrating cell membranes and damaging bacterial cell walls. All the above-mentioned features give this natural biodegradable polymer great potential in a wide range of applications in medicine, the food industry, agriculture, and textiles. Chitosan can be processed into the form of fibres, films, gels, sponges, beads, nanoparticles and micro- and nanofibres. Its antimicrobial activity usually limits or prevents the growth of microorganisms by reducing the growth rate or the number of viable microorganisms [43]. Reports analysing the chitosan market forecast that the global demand for this polymer is steadily growing. In 2019, the chitosan market in the USA was worth approximately USD 6.8 billion [44]. This puts chitosan in second place, after cellulose, in the group of mass-produced natural polymers. Biomaterials based on the aforementioned polysaccharides, to a large extent, meet the requirements of modern dressing materials and implants. The area of application of chitosan, which has attracted great interest for many years, is the production of dressing materials with a broad spectrum of activity [45,46,47]. Most often they are hydrogel materials, based both on neat chitosan and composite materials. Extensive studies have shown that chitosan hydrogel accelerates the process of new blood vessel formation and re-epithelisation in full-thickness skin wounds. Chitosan hydrogels can also be useful in treating deep burns. Kiyozumi et al. [48] compared the effectiveness of a chitosan hydrogel dressing and collagen dressing. In both groups studied, the time required to close the wound was similar, but in the case of chitosan, dressing the thickness of the granulation tissue was greater. In the chitosan group, the process of creating new vessels was also faster, and the layer of the epidermis formed was thicker. The advantage of chitosan dressing was its faster biodegradation compared to collagen dressing, which, according to the authors, was the result of Dulbecco’s Modified Eagle Medium: Nutrient Mixture F-12 (DMEM/F12) used as a substrate. Faster degradation of the hydrogel resulted in a reduction in the number of neutrophils in the wound, which in turn led to a reduction in inflammatory processes and the formation of a thicker epidermis. There are many companies in the world that manufacture various types of dressings based on chitin, chitosan and their derivatives. An example is Eisai Co. (Tokyo, Japan), which produces chitin dressings in the form of a sponge (Chitipack S^®^) as well as non-woven fabric modified with chitin (Chitipack P^®^) or chitosan (Chitopack C^®^). On the other hand, the Japanese company “Unitika” (Osaka, Japan) produces a nonwoven dressing made of chitin fibres, and the American consortium 3M offers chitosan formulations in the form of a gel (TegasorbR) or a hydrocolloid (TegadermR) intended for the treatment of extensive internal wounds [49]. In 2002, the USA Army was equipped with the battlefield chitosan wound dressing HemCon Bandage™ (HemCon, Portland, OR, USA), with haemostatic properties [50]. There are also other hemostatic dressings that use chitin and chitosan derivatives as bioactive agents, e.g., the Syvek patch and RDH (Marine Polymer Technologies, Burlington, VT, USA), Clo-Sur PAD (Medtronic/Scion, Miami, FL, USA), Chito-Seal (Abbott, Redwood City, CA, USA), M-Patch, and Trauma DEX (Medafor, Minneapolis, MN, USA) [51]. In Poland, the only manufacturer of a chitosan-based dressing is TRICOMED S.A., a subsidiary of the dressing materials manufacturer TZMO in Toruń, Poland. Viscoplast (Wroclaw, Poland) is a Polish company belonging to the international consortium 3M, and Urgo Polfa Łódź is a part of the French company Urgo (Chenove, Bourgogne Franche Comte, France). Other producers of dressing materials are Paso-Trading Ltd. (Pabianice, Poland), MaiMed (Tarnowskie Góry, Poland), Ferita (Mokronos Dolny, Poland), Kikgel Ltd. (Ujazd, Poland), and Zarys Ltd. (Zabrze, Poland) The potential and development of the market related to special-purpose dressings are proven by the activity of Polish companies and research centres in this area. It is worth mentioning the hydrogel dressing produced by the MedVentures company (Earth City, MO, USA), the studies of the Institute of Catalysis and Surface Chemistry of the Polish Academy of Sciences in Krakow on a dressing customised for the patient’s needs, and attempts to develop dressings by the Xeno-GP and Prevlly start-ups (Lublin, Poland). Additionally, analysis of the relevant literature indicates the great interest of medical personnel in increasing the effectiveness of the treatment of wounds in patients. According to the needs of the circular economy, the Łukasiewicz Research Network at the Institute of Biopolymers and Chemical Fibers also conducts research work aimed at creating an alternative to polypropylene materials currently used in industry, replacing them with biodegradable polymers. The use of biodegradable materials helps to reduce the costs associated with the storage and disposal of waste, being at the same time a response to global trends in the production of mainly disposable products. According to a report released by the European Environment Agency, nature has also suffered from the COVID-19 pandemic. The increased production and use of medical/protective masks has resulted in greater emissions of greenhouse and other harmful gases and has generated waste that can harm animals and entire ecosystems. Scientists have estimated that during the COVID-19 pandemic, humanity uses about 3 million disposable masks per minute, which amounts to as many as 130 billion masks per month. Assuming that one disposable mask weighs approx. 3.5 g, it turns out that the total weight of masks used every month exceeds 450 t. Therefore, the Łukasiewicz Research Network at the Institute of Biopolymers and Chemical Fibers, in order to protect the environment and reduce the amount of harmful waste, is conducting intensive research on the production and launch disposable masks that are a barrier to pathogens and made of biodegradable polymers. The work carried out so far at the Institute has confirmed such a possibility, and the research is at the stage of determining the time of the biological decomposition of a mask in compost. Moreover, the Łukasiewicz Research Network at the Institute of Biopolymers and Chemical Fibers has produced biodegradable nonwovens intended for agricultural applications. In order to improve the quality and efficiency of crops, work has also been carried out at the Institute on the synthesis of biopolymers for the production of plant pots facilitating plant germination and rooting. Currently, advanced research is also being carried out on the production of food packaging with the use of biodegradable polymers. In the Łukasiewicz Research Network at the Institute of Biopolymers and Chemical Fibres, a team of specialists, in close cooperation with other scientific units, has been dealing for many years with the development of new biomaterials based on polymers of natural and synthetic origin in terms of their use in the prevention and protection of public health. One of the directions of the Institute’s activity is conducting research related to the physico-chemical modification of natural polymers in order to produce new forms thereof (e.g., microcrystalline chitosan, chitosan sponges, films, hydrogels, nanofibres, micro- and nanofibrids) with special properties useful in the construction of multifunctional dressing materials for first aid and for difficult-to-heal wounds, as well as biocomposite materials supporting tissue regeneration processes, including those constituting a basis for the proliferation of stem cells, which also act as a carrier of medicines [52,53]. The solutions developed in the field of implant materials and tissue adhesives based on the selected form of chitosan and hydroxyapatite can be used in the treatment of bone defects in bone surgery [54]. The Łukasiewicz Research Network at the Institute of Biopolymers and Chemical Fibres Institute has ISO 8 class Clean Rooms, which are required for the production of biomaterials for medical, pharmaceutical, and veterinary applications. In Clean Rooms, with the use of specialised equipment, technological processes are carried out for the production of various utility forms of natural polymers.

## 4. Biomaterials Polymers in Łukasiewicz—IBWCh Applications

In the Łukasiewicz Research Network at the Institute of Biopolymers and Chemical Fibres, a dressing material was developed on the basis of natural polymers in the form of a sponge with a quasi-fibrous structure obtained by the freeze-drying method, which is intended to treat wounds at all stages of healing. Chitosan and chitosan–alginate microfibrids with the addition of calcium ions were used for the preparation of the dressing. The material developed in the form of a sponge was characterised by adequate mechanical strength and very good sorption properties and did not show any cytotoxic effect. In in vitro contact with the citrate plasma, chitosan–alginate sponges containing calcium ions activated the plasma coagulation system, thus shortening the coagulation time of the intra- and extrinsic system [55]. The utility forms of natural polymers have also found application in the preparation of various types of implants (e.g., intended for the bridging of damaged peripheral nerves). Such a peripheral nerve prosthesis model was made on the basis of microcrystalline chitosan using the lyophilisation method. The core of the prosthesis was a sponge containing 13 parallel channels, which were intended to guide the growth paths of the damaged nerves and facilitate their anastomosis. To make it easier for a neurosurgeon to sew the implant to the stumps of damaged nerves, the prosthesis core was placed in a suitable sleeve-shaped sheath. The implant model was subjected to in vivo biological tests on animals (Wistar C rats), where a fragment of the sciatic nerve was replaced with the prosthesis developed [56,57]. Histological evaluation of the implanted prosthesis along with proximal and distal fragments of the nerve confirmed a slight inflammation in the form of lymphocytic infiltration. In 89% of animals from the group of operated animals, increased regrowth of nerve fibres was found. At present, world research in the field of polymers shows a trend towards obtaining modern, environmentally friendly biomaterials, thus replacing polyolefins produced from crude oil with natural macromolecules from renewable sources. From a processing point of view, the greatest advantage of synthetic polymers obtained from petroleum (polyethylene, polypropylene) is their thermoplasticity, which enables easy production of various products from polymer melt. Around the world, many attempts are made to modify polysaccharides by physico-chemical methods, aimed at thermal processing without their degradation. This process leads to the plasticisation of the polymer and makes it possible to process it using melt moulding. The production of unique derivatives of natural polymers with improved thermal stability will allow the scope of application of these polymers to increase in the future, e.g., in the packaging industry, tissue engineering, and medicine [58,59,60,61]. Acting towards a circular economy, at Łukasiewicz—IBWCh, a technology for the production of composite surgical meshes intended for tension-free hernia treatment has been developed. Modified with a chitosan coating, OPTOMESH™ Macropore meshes, manufactured by Tricomed S.A. (Lodz, Poland), are characterised by partial resorbability, which allows for faster healing and reduces the stiffening of the implant with in-growing tissue, thereby improving the blood supply to organs located in the vicinity of the implant. The implants manufactured were tested for biocompatibility in accordance with the applicable ISO standards at the Department of Experimental Surgery and Biomaterials Research, Medical University in Wrocław, and at the National Institute of Health in Warsaw. Studies using experimental animals showed a weak cytotoxic effect, but not limiting the possibility of using the modified meshes in surgical procedures, and a lack of irritating or sensitising effects [62,63]. Researchers at Łukasiewicz—IBWCh have modified commercial cellulose products, commonly used in homes and hospitals, with nanoparticles of natural polymers in order to improve their physico-mechanical, sorption, and antimicrobial properties. Microcrystalline chitosan nanoparticles and the chitosan/alginate Na/Ca complex with a particle size of less than 1 μm were used for the modification of cellulose surfaces, with the use of an ultrasonic reactor (Hielscher UP 200S). Modified commercial dressing materials were obtained characterised by a significantly increased absorption capacity, which facilitates the drainage of moisture outside the dressing and provides an optimal environment for wound healing. They also show antibacterial activity against Gram (−) *Escherichia coli and Gram* (+) *Staphylococcus aureus bacteria*, as well as antifungal activity against *Candida albicans* and *Aspergillus Niger* [64,65]. Using biomaterials, a method of preparing collagen-modified chitosan fibres (Chit/Col) from chitosan–collagen solutions was also developed for the construction of scaffolds used in the regeneration of cartilage tissue. Chitosan derived from prawns (*Pandalus borealis*) as well as type 2 and type 3 collagen from calf skin were used to form the fibres. The suitability of chitosan and collagen for the preparation of spinning solutions and Chit/Col fibres was determined. The rheological properties of chitosan–collagen solutions used in spinning Chit/Col fibres with a linear density in the range of 3.30–8.36 dtex were discussed. The fibres obtained were characterised by an increased nitrogen content and strength compared to pure chitosan fibres [66]. As part of the research and development activity, the Łukasiewicz Research Network—Institute of Biopolymers and Chemical Fibres together with the Łukasiewicz Research Network—Institute of Heavy Organic Synthesis “Blachownia” has lately started a project related to the development of biodegradable, plasticised chitosan pellets. The product obtained can be processed by the thermomechanical method and is characterised by significantly extended and different utility properties as compared to the product that is currently obtained as a result of wet processing. The developed plasticised chitosan will be used for the production of special-purpose dressings, so-called occlusive dressings, for the treatment of moderately haemorrhagic wounds and open chest wounds. The Łukasiewicz Research Network at the Institute of Biopolymers and Chemical Fibres is also conducting research on the development of a biopolymer hydrogel composition that is a carrier of active plant extracts to be used on anti-bedsore mats for people undergoing oncological treatment.

## 5. Future Directions of the Development of Biomaterials in the Medical Sector in Relation to the Circular Economy

The transition from synthetic polymers to alternative biological materials with the desired physical and chemical properties of conventional synthetics has attracted the attention of many researchers. Full knowledge of biopolymer networks is essential for the design of future functional materials. This process requires precise control of the physical and chemical properties of the materials used. Improving the properties of biopolymers improves their practical application, not only in the medical industry but also in other industries. The non-toxicity and biodegradability of biopolymers in comparison with synthetic polymers increases their use in, for example, medical devices. The medical sector requires new biomaterials with special functional and performance properties, which, when subjected to appropriate modification of their chemical and phase composition, will achieve a high limit of biocompatibility. It is expected that in the near future there will be an increase in the use of conductive polymers, hybrid materials and composite materials produced with the use of nanotechnology. Research is being carried out on methods of covering the surfaces of artificial organs with polymer layers, with the possibility of releasing drugs coated with ceramic or metallic layers (e.g., titanium compounds or carbon nanolayers) to improve biocompatibility and long-term wear resistance as well as biodegradation properties. New-generation polymeric materials are increasingly used in tissue engineering, reconstructive surgery, and chemotherapy. Research on silk tailoring is being carried out through genetic engineering that takes into account specific chemical characteristics that will aid drug and gene delivery [67]. Biopolymers have the ability to be functionalised with specific ligands and to encapsulate hydrophobic and hydrophilic drugs to achieve controlled and targeted drug release at the desired site of action [68]. In scientific centres around the world, research is carried out on the possibility of creating tissues using stem cells obtained in various ways. Stem cells constitute great hope of modern medicine, and introducing them into the body will contribute to the regeneration of organs. As a result of the application of methods using natural or synthetic polymer matrices, using knowledge of a given organ, its mechanics, and its physiology, it will be possible to create fully functional organs in in vitro culture that are able to function properly in a patient’s body.

An important research direction in the medical sector is molecular diagnostics, which uses methods of molecular biology, chemistry, and biophysics to identify changes in the structure of genetic material and in the gene transcription profile, as well as differences in the set of proteins in cells and tissues. Due to knowledge of the structure of all human genes, global genetic analysis (DNA analysis), transcriptomic analysis (RNA analysis), and proteomic analysis (protein analysis) are possible. As part of preventive measures, these methods will enable the identification of people at increased risk of developing a specific disease, as well as its earlier detection, more accurate diagnosis, and monitoring of treatment progress. The use of molecular diagnostic tools in clinical practice will entail an intensive development of scientific research in the fields of nanomedicine, nanobiotechnology, molecular biology, and bioinformatics. An interesting technology in the field of biomaterial research is gene therapy, which enables the repair or replacement of mutated fragments of genetic material in people whose gene defect is the cause of serious, even life-threatening pathological changes. The repair of the genetic material can be accomplished by making back-corrective mutations, introducing a correct copy of a specific gene into cells, or replacing a defective copy of a gene with a correct gene. Genotherapy can be combined with cell therapy, including stem cell therapy. Improving the methodology of genotherapy will also stimulate the development of pharmacogenomics, targeted therapies, anti-angiogenic therapies, polymer carrier chemistry, pharmacology, immunology, and regenerative medicine based on the use of stem cells [69]. Biopolymers are also widespread mainly due to their use as medical implants in tissue engineering or regenerative devices. Potential therapeutic applications of biopolymers—bioimplants include the engineering of cardiac, vascular, bone, cartilage, and nervous tissue, as well as the matrix of a drug delivery vehicle [70]. The development of new solutions based on biomaterials and biopolymers will dominate in the near future due to environmental benefits in the field of sustainable development.

## 6. Summary

The growing problem of the disposal of waste has made biomaterials a more and more common alternative to the commonly used, difficult-to-recycle plastic. Environmental protection is critical for the future of humanity. The application of biomaterials containing biodegradable polymers is undoubtedly a very important element of environmental protection. Biomaterials are used in many branches of the economy, but the dominant one is the medical industry, mainly associated with the production of specialised dressing materials. Biomaterials are used extensively in the design of modern dressing kits, implants, surgical sutures, stents, and craniofacial anastomoses. The use of biomaterials to design new materials for various applications is a common research topics throughout the world. However, there are still many problems regarding biomaterial application, and there is actually a long way to go to completely solve all arising problems.

## Data Availability

Not applicable.
